# Frontiers in stimuli-responsive nanoplatforms: pioneering drug delivery in nanobiotechnology

**DOI:** 10.1039/d4na90074j

**Published:** 2024-07-12

**Authors:** Juan C. Cruz, Luis H. Reyes

**Affiliations:** a Department of Biomedical Engineering, Universidad de los Andes Bogotá Colombia jc.cruz@uniandes.edu.co; b Department of Chemical and Food Engineering, Universidad de los Andes Bogotá Colombia lh.reyes@uniandes.edu.co

## Abstract

Prof. Juan C. Cruz and Prof. Luis H. Reyes introduce the *Nanoscale Advances* themed issue on Frontiers in Stimuli-Responsive Nanoplatforms: Pioneering Drug Delivery in Nanobiotechnology.
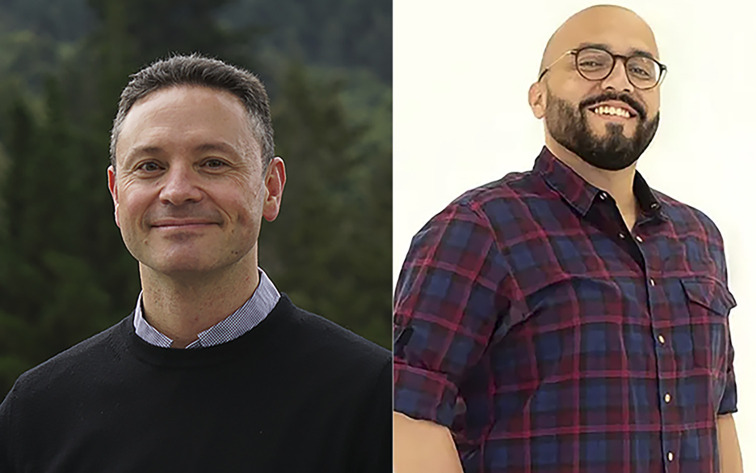

The rapid progress in technology has led to the merging of nanotechnology and biomedicine, which is creating new opportunities for therapeutic interventions. This is especially true in the field of stimuli-responsive nanoplatforms, which is breaking new ground. The collection titled “Frontiers in Stimuli-Responsive Nanoplatforms: Pioneering Drug Delivery in Nanobiotechnology,” curated by Professors Juan C. Cruz and Luis H. Reyes, presents a diverse selection of review and original research articles that demonstrate the significant capabilities of these advanced materials in transforming drug delivery systems.

The combination of stimuli-responsive technology and nanobiotechnology revolutionizes medicine by allowing accurate targeting and timing of therapeutic interventions. These adaptable systems react to specific stimuli—whether they are biological, pharmacological, or physical—ensuring targeted, regulated, and patient-focused drug delivery. This collection presents groundbreaking research in various fields, ranging from overcoming neurological obstacles through the use of advanced vector technologies to improving the effectiveness of photothermal cancer treatments utilizing customized nanoparticles.

Prominent features of the collection include pioneering research, such as the creation of dual-color core–shell silica nanosystems by Ramirez-Morales *et al.*, which provide potential advancements in super-resolution biomedical imaging (https://doi.org/10.1039/D3NA00310H). This technique incorporates fluorescent nanoparticles that have the potential to completely transform diagnostic procedures and allow for the continuous monitoring of biological activities at levels that have never been achieved before. The researchers successfully produced dual-color fluorescent core–shell silicon dioxide nanoparticles using a two-cycle Stöber process. They further enhanced the colloidal stability of the nanoparticles by functionalizing them with biotin. The nanosystems underwent thorough analysis utilizing a range of analytical and sophisticated microscopy techniques, which uncovered their promise as versatile instruments for fluorescence optical nanoscopy and biological applications.

In the insightful study “light-initiated aggregation of gold nanoparticles for synergistic chemo-photothermal tumor therapy”, presented by Xia *et al.*, the researchers explore a novel therapeutic strategy integrating chemotherapy with photothermal therapy using light-responsive gold nanoparticles. These nanoparticles are designed to aggregate when exposed to laser light, significantly enhancing the localized delivery and effectiveness of the chemotherapeutic drug doxorubicin. The controlled aggregation at the tumor site ensures precise drug release and amplifies the photothermal effect, resulting in notably improved tumor reduction. This innovative approach highlights the transformative potential of combining advanced nanoparticle engineering with targeted cancer treatment strategies, setting a new benchmark for synergistic cancer therapies.

The thematic issue also explores the complexities of gene delivery systems that specifically target the central nervous system. Gao discusses techniques to overcome the challenging blood–brain barrier using advanced vector technologies and remote-control technologies to regulate the extracellular space (https://doi.org/10.1039/D3NA01125A). The review examines the impact of the extracellular space on the movement of drugs and emphasizes the importance of using remote-control technology to aid in crossing the blood–brain barrier. In addition, the author explores the most recent developments in delivering therapeutic gene editing directly to the central nervous system (CNS) tissue. The author also examines the influence of artificial intelligence, machine learning, ultrasmall soft endovascular robots, and high-resolution endovascular cameras on enhancing gene delivery to the CNS.

Singh *et al.* conduct a comprehensive analysis of MCM-41 derived berberine-loaded porous silica nanoparticles, with a specific emphasis on their ability to hinder the apoptotic process in neuronal cells (https://doi.org/10.1039/D3NA01142A). The authors utilize a modified Stöber approach to create nanoparticles that have uniform size and contain a large amount of medication with excellent efficiency in trapping it. The study demonstrates that nanoparticles have the capacity to enhance mitochondrial health, revive cellular viability, and inhibit the apoptotic process in SH-SY-5Y cells. This research provides crucial information on the production and potential of nanoparticles as an effective therapeutic intervention for illnesses related to apoptosis.

The collection also examines the engineering elements of nanoparticle design, with Riahi *et al.* investigating the impact of surfactant ratios on the production of single-core iron-based nanoparticles for magnetic hyperthermia and their absorption by endothelial cells (https://doi.org/10.1039/D3NA00540B). The authors illustrate that the simultaneous utilization of oleic acid and oleylamine as surfactants leads to a significant saturation magnetization and improved heating rate for magnetic fluid hyperthermia. The process of modifying the oleic acid-coated nanoparticles with trimethoxysilane results in the creation of a core–shell structure that displays exchange bias. The effective absorption of these nanoparticles by human umbilical vein endothelial cells demonstrates their potential for use in biomedical applications.

Vergaro *et al.* utilize chemical synthesis and nanotechnology to develop a versatile platinum drug delivery system using a unique metal complex containing the bioactive compound curcumin (https://doi.org/10.1039/D3NA00200D). This method exhibits enhanced bioavailability and therapeutic efficacy. The authors utilize ultrasonication in conjunction with layer-by-layer technology to create nanocolloids that possess advantageous characteristics like excellent biocompatibility, increased solubility in water-based solutions, stability, high drug capacity, and improved biological effectiveness compared to the unbound drug. The study demonstrates the potential of this technique in altering the bioavailability of platinum-based medicines and enhancing their therapeutic efficacy in terms of both cytotoxic and anti-metastasis effects.

Basaran *et al.* conducted a study on the pH-dependency of DC-SIGN/R multivalent lectin-glycan interactions utilizing polyvalent glycan-gold nanoparticles (https://doi.org/10.1039/D3NA01013A). Their findings revealed unique pH-dependent binding behaviors and their possible significance in virus binding and release processes. Jirát-Ziółkowska *et al.* present evidence of the extended *in vivo* degradation of injectable polymer implants that are responsive to changes in temperature and pH (https://doi.org/10.1039/D4NA00212A). These implants can be traced using ^19^F magnetic resonance imaging, providing a promising platform for long-term localized theranostics. Tricase *et al.* describe the creation of bionanoreactors based on apoferritin, which are activated by bioelectrochemical means (https://doi.org/10.1039/D3NA01046E). These bionanoreactors are used for the synthesis and monitoring of CdSe nanoparticles, and they incorporate leaky waveguides. The study demonstrates a unique method for producing semiconductor nanoparticles of specific sizes by utilizing a protein-based bionanoreactor that is activated by pH changes at the interface between the electrode and solution.

In addition, the collection includes a compelling study conducted by Gamal *et al.*, which examines the effectiveness of photothermal therapy aided by gold nanorods in treating breast tumors in adult female rats (https://doi.org/10.1039/D3NA00595J). The authors utilize a rat model that is induced with 7,12-dimethylbenz[*a*]anthracene (DMBA) to evaluate the effectiveness of a therapeutic technique that combines polyvinylpyrrolidone-capped gold nanorods and near-infrared laser irradiation. The work reveals the groundbreaking impact of this photothermal therapy method in specifically eliminating breast cancer cells, providing a hopeful pathway for future cancer treatments.

The collection provides a complete overview of the current state and future possibilities of stimuli-responsive nanoplatforms by combining various studies. It emphasizes both the achievements of individuals and the collective endeavors of the worldwide research community, whose commitment to advancing the boundaries of scientific potential ensures a future when nanotechnology-based treatments are widespread.

This editorial collection serves as a repository of knowledge and a source of inspiration to drive further innovation and encourage interdisciplinary collaborations. These collaborations are essential for the advancement of these technologies into clinically relevant solutions as we progress in our research and development. The research described in this work not only enhances our comprehension of the underlying principles that regulate stimuli-responsive nanoplatforms, but also offer practical knowledge regarding their design, synthesis, and application in different biomedical scenarios.

We extend our utmost appreciation to all authors for their exceptional contributions, the anonymous reviewers for their valuable time and expertise in assessing the submissions, and the editorial staff at *Nanoscale Advances*, particularly Dr Hannah Kerr, for their guidance and support during the preparation of this issue. Their combined endeavors have played a crucial role in moulding this compilation into a significant asset for the scholarly community.

Essentially, “Frontiers in Stimuli-Responsive Nanoplatforms” serves as both a demonstration of scientific quality and a guiding force in the pursuit of advanced, effective, and precise drug delivery systems. We encourage readers to fully engage with this collection, as we are confident that it will provide valuable insights and stimulate new avenues of study and partnerships that will continue to advance the field of nanobiotechnology. In this pivotal moment of advancing therapeutic interventions, this compilation acts as a guiding light, shedding light on the direction towards a future when tailored, accurate, and patient-focused treatments are not merely a potentiality, but an actuality.

